# Role of DTL in Hepatocellular Carcinoma and Its Impact on the Tumor Microenvironment

**DOI:** 10.3389/fimmu.2022.834606

**Published:** 2022-03-22

**Authors:** Zuyin Li, Rangrang Wang, Chen Qiu, Can Cao, Jianming Zhang, Jun Ge, Yuanping Shi

**Affiliations:** ^1^ Department of Hepatobiliary Surgery, Peking University Organ Transplantation Institute, Peking University People’s Hospital, Beijing, China; ^2^ Department of General Surgery, Shanghai General Hospital, Shanghai Jiao Tong University School of Medicine, Shanghai, China; ^3^ Department of General Surgery, Huadong Hospital Affiliated to Fudan University, Shanghai, China; ^4^ Center of Gallbladder Disease, Shanghai East Hospital, Institute of Gallstone Disease, School of Medicine, Tongji University, Shanghai, China; ^5^ Department of General Surgery, The 306th Hospital of People's Liberation Army (PLA)-Peking University Teaching Hospital, Beijing, China; ^6^ Department of Endocrinology, Shanghai Ninth People’s Hospital, Shanghai Jiao Tong University School of Medicine, Shanghai, China

**Keywords:** DTL, DNA replication, cell cycle, immune cell infiltration, prognosis

## Abstract

**Background:**

The crucial role of *DTL* has been previously implicated in genomic stability; however, its prognostic value and its relation with tumor immunity in hepatocellular carcinoma (HCC) remain to be further explored.

**Methods:**

Transcriptional and mutational datasets as well as clinical information were retrieved from the GEO, ICGC, and TCGA databases. Differentially expressed genes (DEGs) were obtained from the comparison of DTL^high^ and DTL^low^ expression groups of the TCGA-HCC cohort. Those genes were under KEGG and gene ontology (GO) analyses to decipher the influence of the DTL gene on the biological behavior of HCC tumor cells. The survival status and mutational characteristics of patients according to DTL levels were depicted and analyzed. The DTL overexpression in HCC and its impact on prognosis were further confirmed by a cohort of 114 HCC patients (validation cohort). The TIMER, GEPIA, and TISIDB databases were adopted to investigate the potential relations between DTL levels and the status of immune cells, as well as immune cell infiltrations.

**Results:**

The *DTL* gene is overexpressed in tumor tissues compared with distant non-malignant liver tissues, and *DTL* overexpression in HCC would enhance the HCC cells in the activities of cell cycle and division. HCC patients with high *DTL* expression have unfavorable clinical outcomes and harbor more somatic mutations than those with low DTL expression, and multivariate analysis also revealed that *DTL* overexpression could act as an independent biomarker for prognosis. Moreover, the *DTL* gene was positively linked to marker sets of infiltrating activated CD8+ and CD4+ T cells; however, these cells demonstrated to be functionally exhausted.

**Conclusions:**

Patients with a *DTL* overexpression phenotype in HCC have poorer prognosis than those in the DTL^low^ group due to the role of the *DTL* gene in the process of pro-cell proliferation, accompanied by the immunosuppressive microenvironment and T cell exhaustion.

## Introduction

Primary liver cancer is the 6th most common cancer worldwide and the 3rd leading cause of cancer-related death, second only to lung cancer and colon cancer. The number of hepatocellular carcinoma (HCC) cases takes up approximately 75%–85% of primary liver cancer; the rest were intrahepatic cholangiocarcinoma (ICC) (10%–15%) and other rare types. According to statistics, in 2020, there were 905,677 new cases diagnosed with liver cancer and 830,180 cases of liver cancer-related death worldwide (1). China is one of the areas with high incidence of HCC and whose cases in China account for more than half of the global liver cancer patients, and it is one of the areas with the heavy medical burden of HCC diseases (2). In view of the increasing incidence rate of HCC in China and other countries in the world, finding markers that can indicate the clinical outcomes of liver cancer and understanding the course of disease are helpful in finding potential therapeutic targets.

Compared with other cancer types, the therapeutic effects of advanced HCC, either surgical resection or chemo, were rather unsatisfactory, which remained a difficulty in tumor treatment (3). New systemic therapies (combined targeted therapy and immunotherapy) soon become the main treatment for advanced liver cancer (3). Undoubtedly, new therapies targeting immune or cancer cells have raised hope for patients; however, not all patients showed a lasting response to such methods due to the tumor immune heterogeneity and immunotherapy sensitivity (4). The population responding to immunotherapy in HCC is limited, which may be associated with the small number of invasive T cells or killer cells in the tumor, the so-called cold tumor (5). It is the bottleneck and key to screening patients with hot tumor characteristics for immune therapy. Therefore, researchers were looking for genes related to tumors with hot/cold characteristics and could reflect the immune status of patients. These genes may hopefully help to screen patients who would possibly respond to immunotherapy. In this study, we mainly discussed the *DTL* gene to explore its prognostic value, biological impact, and role, as an indicator, in cold–hot characteristics in HCC.

*DTL* (denticleless protein homolog), also known as *CDT2* gene, which contains multiple WD40-repeat domains plays a crucial role in regulating the degradation of CDT1 after DNA damage (6). Previous studies have shown that the CRL4^CDT2^ complex, together with Rad6/18, monoubiquitinated PCNA to promote the translesion DNA synthesis in undamaged cells (7). In addition, the CRL4^CDT2^ complex can degrade DNA replication-related proteins in a proteasome-dependent manner during DNA replication, implying a crucial role of DTL (CDT2) in the regulation of DNA replication (8). Others have shown that DTL (CDT2) is augmented in head and neck squamous cell carcinoma (HNSCC) and is necessary for those tumor cells to proliferate. Its main role is to inhibit abnormal DNA replication. Inactivation of CRL4^CDT2^ enhances the radiosensitivity of HNSCC cells (9). Furthermore, some scholars have reported that USP46 protein could mediate the stability of DTL (CDT2) and promote the growth of HPV-positive tumors, suggesting the potential role of *DTL* in tumor growth (10). Considering the close associations between *DTL* gene, DNA replication process, and tumor progression, we wanted to know whether the *DTL* gene played an undefined or otherwise similar role in liver cancer like other tumor types.

The present study investigated the levels of *DTL* expression in HCC and its association with prognosis in the HCC cohort and explored the relation of *DTL* expression with infiltrating immune cells as well as markers of immune status *via* multiple databases. The results pinpointed the crucial role of the *DTL* gene in the progression of HCC and illustrate a relationship between DTL expression and the clinical outcome of the HCC cohort as well as immune cell infiltration.

## Materials and Methods

### Data Resources and Descriptions

Transcriptional profiles of five independent cohorts, containing tumor tissues and paired non-tumor specimens, were summarized from the Gene Expression Omnibus (GEO) database. The detailed information of each cohort is listed in **Supplementary Table 1**. TCGA-LIHC mRNA expression profiles and data of clinicopathological characteristics in each subject were downloaded from the TCGA database (https://cancergenome.nih.gov/). The TCGA-LIHC cohort contains 421 specimens, with 371 primary tumor and 50 normal liver samples. The RNA-seq data of the ICGC-LIHC-JP cohort contains 240 primary tumor tissues and 202 non-tumor tissues, which were summarized from the ICGC database (https://dcc.icgc.org). The “edgeR” package was utilized to evaluate the *DTL* expression between tumor and non-tumor tissues.

To demonstrate the effects of DTL knockdown on the process of cell proliferation, we analyzed the GSE20357 data set, which contained transcriptional profiles of DTL knockdown data of three Ewing’s sarcoma (ES) cell lines. To explore the therapeutic potential of pevonedistat (MLN4924) on tumor cells, two data sets, GSE30531 and GSE184850, contain the transcriptome of two types of cell lines after the treatment of the drug. We summarized the impact of MLN4929 on key genes related to cell cycle, DNA replication, and apoptosis.

### Validation Cohort

To validate the bioinformatic results, we obtained 114 cases of HCC to form a validation cohort. The cohort consisted of 114 patients who were confirmed with HCC in the Department of General Surgery, Shanghai General Hospital. The clinical data of these enrolled subjects were collected from electronic medical records. All samples collected after surgery were pathologically confirmed and fixed in 4% paraformaldehyde. This study was approved by the Ethical Committees of Shanghai General Hospital, and all procedures were strictly executed by the principles of the Declaration of Helsinki, and written informed consent was obtained from each subject.

### Histology and Staining

The paraffin-embedded samples after surgery were deparaffinized with conventional protocols and rehydrated for immunohistological (IHC) examination according to a standard protocol. Primary antibodies anti-CDT2 (1:500, #ab72264, Abcam) were used. Images were scanned by a Leica microscope (Germany).

### Differentially Expressed Gene Analysis

We extracted the differentially expressed genes (DEGs) between two groups of patients (DTL^high^ and DTL^low^ groups) identified according to the median counts of DTL expression by applying the “edgeR” package. Log2(Fold change)>1 and adjusted p value < 0.05 were deemed as the standard to screen DEGs. To explore the functions of the 839 upregulated DEGs, those genes were further analyzed *via* DAVID 6.8 for KEGG pathway and GO functional analyses (https://david.ncifcrf.gov/). The correlations of the *DTL* gene with cell cycle- or DNA replication-related genes were calculated by the function “cor.test” based on the R studio platform. The dot plot was generated by the function “ggcatterstats” included in “ggstatsplot” package, and results also were subject to Cytoscape 3.5.1 for visualization in ClueGO modules.

### Kaplan–Meier Plotter Database Analysis

The Kaplan–Meier plotter could evaluate the effect of the DTL gene in various cancer types, including hepatocellular carcinoma (n = 364). Sources for the database come mainly from TCGA. The liver cancer module of the database was applied to explore the association of DTL levels with the survival of the HCC cohort (http://kmplot.com/analysis/) (11). The plot contains the value of hazard ratio (HR) with 95% confidence intervals and log-rank p-values.

### TIMER Database Analysis

TIMER is a web tool for the integral analysis of immune infiltration across various tumor types (https://cistrome.shinyapps.io/timer/) (12). The tool employs a deconvolution algorithm to estimate the levels of tumor-infiltrating immune cells (TIICs) based on transcriptional profiles (13). The DiffExp module was used to evaluate the levels of DTL expression in diverse tumor types, and gene modules were applied to dissect the association of DTL expression and immune infiltration. The correlation module was utilized to evaluate the relationship of DTL levels with markers of TIICs. These markers or immune status of TIICs were referenced in prior studies (14–16).

### Gene Correlation Analysis in GEPIA

GEPIA is another web tool which analyzes the RNA-sequencing expression of diverse tumor types (17). In our study, this database was applied to further confirm the associations of *DTL* expression with marker sets of TAMs and monocytes. Pearson correlation analysis was applied to evaluate the correlation coefficient of *DTL* with other interested genes. The R and *P* values for the correlation between DTL and interested genes calculated by GEPIA are summarized in **Supplementary Table 3**.

### Gene Set Enrichment Analysis

To identify underlying pathways between *DTL*-high and -low groups functioning in HCC, we analyzed relevant pathways using gene set enrichment analysis (GSEA). The median expression of the *DTL* gene acted as a cutoff value to split the enrolled patients into *DTL* ^high^ and *DTL* ^low^ groups. All genes were sequenced based on their differential expression between the two groups. The normalized enrichment score (NES) of specific pathways was evaluated, and each analysis performed 1,000 times of gene set permutations. A *p*-value < 0.05 was deemed statistically significant.

### Somatic Mutation Analysis

Among 371 cases of HCC patients, 364 cases with mutation data were stored in the TCGA-GDC database (TCGA.LIHC.mutect.somatic.maf.gz). We cut the mutation profiles into two groups (DTL^high^ and DTL^low^ groups) according to the transcriptional levels of DTL gene in the original cohort. The function “oncoplot” in the “maftools” package on the “R studio” platform was used to draw all and respective mutation maps of the two groups, and the mutant genes with significantly different distribution were evaluated *via* the “mafCompare” function in the “maftools” package.

### Statistical Analysis

The differences between transcriptional levels of two defined groups were based on Student’s t-test or the Mann–Whitney U-test. The relationship of *DTL* levels with clinicopathological parameters was estimated by applying the chi-squared test. SPSS version 23.0 statistical software from SPSS Inc. (Chicago, IL, USA) was used across this study. *p* < 0.05 was deemed statistically significant.

## Results

### Upregulated DTL Expression in Tumor Samples Forebodes Unfavorable Prognosis of HCC Patients

**Figure 1:**
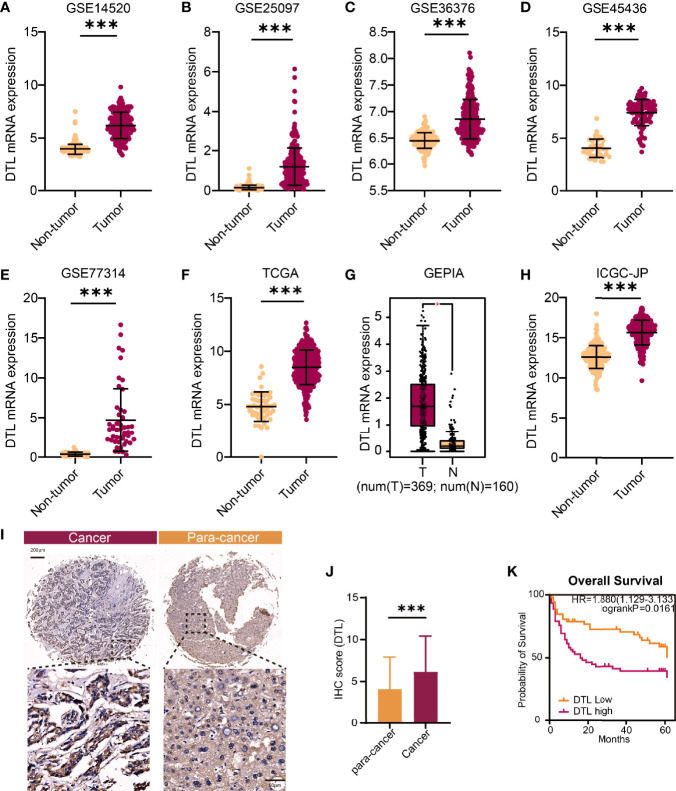
The upregulated *DTL* expression in HCC patients. **(A–F, H)** The comparison of DTL transcriptional levels between tumor and nontumor tissues of HCC patients by Student’s t-test or Mann–Whitney U test based on GEO, TCGA, and ICGC databases. **(G)** Box plot of DTL expressions in tumor and non-tumor tissues from the GEPIA database. **(I, J)** DTL IHC staining and statistical results reveal the protein levels of DTL in the validation cohort. **(K)** OS of the validation cohort stratified into DTL-high and DTL-low groups. *p < 0.05, ***p < 0.001.

To compare the transcriptional expression of the *DTL* gene between tumor and non-tumor-paired tissues, five GEO RNA-seq datasets and RNA sequencing profiles from the ICGC and TCGA databases containing HCC tumor and paired adjacent non-tumor specimens were used. The results revealed that the mRNA levels of the *DTL* gene in tumor samples were significantly elevated in these 6 cohorts from the GEO and TCGA databases (all *p* < 0.01, **Figures 1A**–**E**). Furthermore, the GEPIA database also confirmed that the transcriptional levels of the *DTL* gene in tumor tissues (T, rosaceous) were higher than those in non-tumor tissues (N, orange, **Figure 1G**). The overexpression phenotype of DTL was also confirmed in the ICGC-JP cohort, which contains transcriptional profiles of HCC tumor tissues and distant non-malignant samples (**Figure 1H**). A higher expression of the DTL gene in protein levels was also demonstrated in our own validation cohort (**Figures 1I, J**). Next, we explored *DTL* levels in the TIMER database, which summarized RNA-seq data of multiple tumor types from TCGA. The mRNA levels of DTL were significantly elevated in liver tumor tissues than in adjacent normal tissues and, interestingly, demonstrated a pan-cancer upregulation characteristic (**Supplementary Figure 1A**), implying the crucial role of DTL in tumorigenesis and progression.

**Figure 2:**
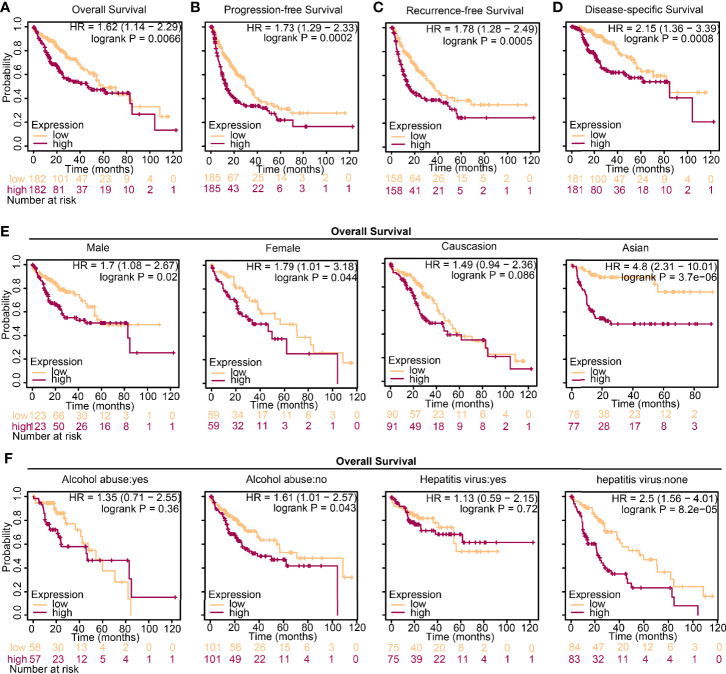
High expression of DTL predicts worse prognosis in HCC. **(A–D)** Survival analysis of HCC patients from the TCGA database grouped by *DTL* expression. **(E, F)** Overall survival (OS) analysis of the effect of DTL expression on the HCC cohort subgrouped by gender, ethnics, alcohol abuse, and infection of hepatitis virus.

To investigate the influences of high-level DTL expression on the HCC cohort, we next investigated the correlations between clinicopathological features and *DTL* levels in the TCGA cohort. For the cutoff by the median expression of *DTL* levels, we split patients equally into two groups (DTL^high^ and DTL^low^). The results revealed a significant clinical relevance of the DTL overexpression phenotype with T stage and pathologic stage (**Table 1**, all *p* < 0.05), suggesting that the *DTL* gene most likely serves as a pivotal role in tumor progression and this speculation was also evidenced by the correlation of DTL expression with tumor size in the validation cohort (**Supplementary Table 2**). Considering that the mRNA levels of *DTL* showed clinical relevance with the survival status of follow-up patients in the chi-square test (**Table 1**, bottom, *p* = 0.018), we next wondered whether the *DTL* overexpression affects the clinical prognosis of the HCC cohort. Survival analysis showed that subjects with DTL overexpression had unfavorable survival in the validation cohort (**Figure 1K**) and in the TCGA-HCC cohort, regardless of overall survival (OS), progression-free survival (PFS), recurrence-free survival (RFS), or disease-specific survival (DFS, all *p* < 0.05) (**Figures 2A**–**D**). Survival analysis for subgroups was also performed in the HCC cohort. Results showed that a high level of *DTL* expression led to worse OS and DFS in male HCC patients, and Asian HCC patients had worse OS and DFS in the DTL^high^ group than those in the DTL^low^ group (**Figure 2E** and **Supplementary Figure 2A**). Moreover, a high *DTL* level significantly contributed to reduced OS and DSS in patients without a record of alcohol consumption or hepatitis virus infection (**Figure 2F** and **Supplementary Figure 2B**). Multivariable Cox regression, after univariate analysis, showed that the elevated expression of the *DTL* gene is an independent and significant risk prognostic factor for OS in patients with HCC (HR = 2.304, *p* = 0.001) after equilibrating the impact of age, T stage, M stage, and AJCC stage (**Table 2**). Collectively, our results suggest that upregulated *DTL* expression predicts poorer prognosis of HCC patients.

**Table 1:** Clinicopathological characteristics in relation to DTL expression status in the TCGA cohort.

Characteristics	TCGA cohort (N = 371)	DTL expression		χ^2^	*p* value
		High	Low		
		N = 186 (%)	N = 185 (%)		
Age					
≥70 y		35 (42.2)	48 (57.8)	2.714	0.099
<70 y		151 (52.4)	137 (47.6)		
Gender					
Male		119 (47.6)	131 (52.4)	1.970	0.160
Female		67 (55.4)	54 (44.6)		
Race					
ASIAN		92 (58.2)	66 (41.8)		
BLACK or AFRICAN AMERICAN		9 (52.9)	8 (47.1)		
WHITE		83 (45.1)	101 (54.9)	12.496	0.014
AMERICAN INDIAN or ALASKA NATIVE		1 (50)	1 (50)		
NA		1 (10)	9 (90)		
T					
T1–T2		128 (46.5)	147 (53.5)		
T3–T4		58 (62.4)	35 (37.6)	9.998	0.007
NA		0 (0)	3 (100)		
N					
N0		134 (53.2)	118 (46.8)		
N1		3 (75)	1 (25)	4.526	0.104
NX		66 (57.4)	49 (42.6)		
M					
M0		139 (52.3)	127 (47.7)		
M1		1 (25)	3 (75)	2.341	0.310
MX		46 (45.5)	55 (54.5)		
Pathologic stage					
Stages I–II		117 (45.5)	140 (54.5)	8.456	0.015
Stages III–IV		57 (63.3)	33 (36.7)		
NA		12 (50)	12 (50)		
Status					
Alive		110 (45.6)	131 (54.4)	5.550	0.018
Dead		76 (58.5)	54 (41.5)		

Statistical significance was determined by the chi-square test or Fisher’s exact test.

**Table 2:** Univariate and multivariate Cox logistic regression analyses of OS in the TCGA cohorts.

Covariates	Univariate analysis			Multivariate analysis		
	HR	95% CI	*p* value	HR	95%	*p* value
Age (ref. ≤70 y)	1.520	1.050–2.200	**0.027**	1.879	1.154–3.061	**0.011**
Gender (ref. female)	0.815	0.572–1.161	0.257			
pT stage (ref. T1–T2)	2.539	1.784–3.611	**<0.001**	2.474	0.333–18.376	0.376
pN stage (ref. N0)	1.999	0.490–8.161	0.334			
pM stage (ref. M0)	3.977	1.250–12.652	**0.019**	4.235	1.207–14.851	**0.024**
AJCC stage (ref. I–II)	2.448	1.689–3.548	**<0.001**	0.985	0.133–7.310	0.988
DTL expression (ref. low)	1.819	1.281–2.583	**0.001**	2.304	1.423–3.730	**0.001**

P values with statistical significance (P <0.05 ) are marked bold.

### HCC Cells With *DTL* Overexpression Showed Pro-Proliferation Characteristics

**Figure 3:**
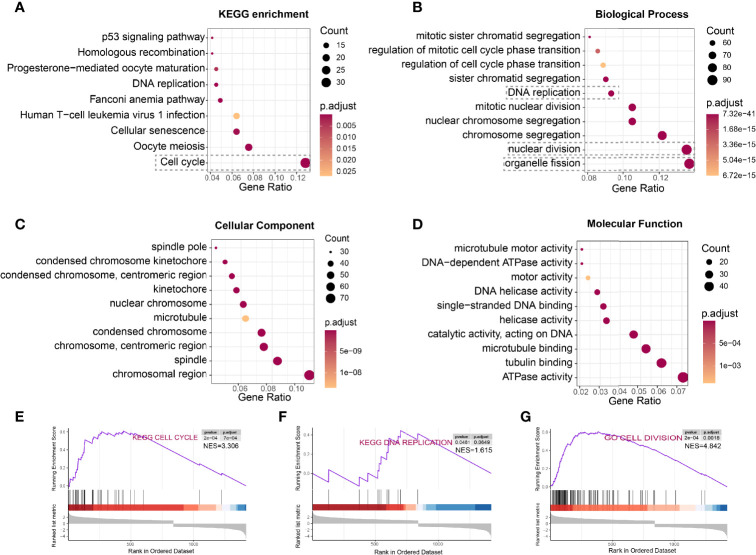
HCC cells with *DTL* overexpression showed pro-proliferation characteristics. **(A–D)** KEGG pathway enrichment and GO analysis of 839 upregulated DEGs. **(E–G)** Gene set enrichment analysis (GSEA) prompts *DTL* is positively related to cell cycle, DNA replication, and cell division.

To determine the underlying function of *DTL* in tumor progression, we calculated the differentially expressed genes (DEGs) in the TCGA-HCC cohort between DTL^high^ and DTL^low^ groups. KEGG enrichment and gene ontology (GO) analyses were utilized to dissect the function of these genes. KEGG enrichment analysis showed that the 839 upregulated DEGs were primarily enriched in the cell-cycle process (**Figure 3A**). Biological processes in relation to these enhanced DEGs were organelle fission, nuclear division, and DNA replication, processes that were related to cell growth and proliferation (**Figure 3B**). Cell components and molecular functions of these upregulated DEGs were also mainly about the preparation for the processes of cell proliferation and division (**Figures 3C, D**). However, the downregulated DEGs were primarily correlated with metabolic pathways, e.g., retinol metabolism and drug metabolism, and the enriched biological processes included small-molecule catabolic process (**Supplementary Figures 3A, B**), suggesting that the metabolic function of HCC cells with high DTL expression was much less alike normal hepatocytes and functionally defected. GSEA analysis was also employed to pinpoint the signaling pathways markedly associated with the mRNA levels of the *DTL* gene (*DTL* ^high^ vs. *DTL* ^low^). The results indicated that the expression profiles of the cohort with high *DTL* expression were enriched in the cell cycle, DNA replication cell division, chromosome segregation, DNA replication initiation, and p53 signaling pathway (**Figures 3E**–**G** and **Supplementary Figures 3C**–**E**), which were similar to the results of KEGG and GO analyses. Due to the lack of DTL-knockdown transcriptional profiles on HCC cell lines (e.g., HepG2 and Huh7), we retrospectively found that researchers knocked down DTL in other tumor cells [three Ewing’s sarcoma (ES) cell lines, (GSE20357)] (18). After the downregulation of the DTL gene, the proliferation-related genes of tumors cells were significantly inhibited and the expression of apoptotic genes increased (**Supplementary Figure 4**).

**Figure 4:**
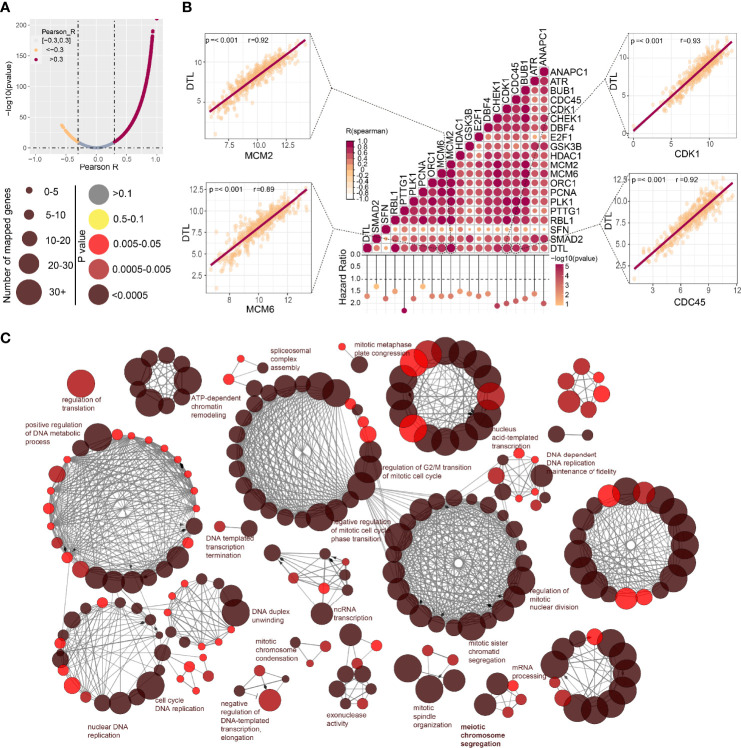
Associations between *DTL* and key genes related to cell cycle in HCC. **(A)** The spoon-shaped plot shows genes related to DTL expression calculated by Pearson correlation analysis (orange dot, R < -0.3; rosaceous dot, R > -0.3). **(B)** Correlation between cell cycle-related genes and DTL, and their impact on prognosis of the TCGA-HCC cohort. **(C)** Visualization of the interaction network of genes strongly associated with DTL by Cytoscape (ClueGO module). Node size indicates the mapped gene number; the node color schedule represents the *p* value.

We next perform co-expression analysis to characterize the genes correlated with *DTL* expression in the HCC cohort and then analyzed those strongly (R > 0.3, p < 0.05) associated with *DTL* expression (**Figure 4A**). We found that cell cycle-related genes, say, CDK1, MCM2, CDC45, and MCM6, showed significant positive associations with DTL expression (all r > 0.8, p < 0.05, **Figure 4B**), and these cell cycle-related genes, which show positive correlations with the *DTL* gene, were mostly risk factors for HCC patients (**Figure 4B**). Meanwhile, the interactive network of these co-expression genes was visualized through ClueGO to demonstrate the biological processes and associations of functionally grouped genes. The interaction networks of these co-expressed genes, which included 406 representative terms and 1,711 term connections, showed that the significantly related biological functions were the cell cycle, DNA replication, and processes that have been considered necessary preparations for tumor growth (**Figure 4C**). Together, this evidence revealed that HCC cells with *DTL* overexpression showed pro-proliferation characteristics.

### Relationship Between Somatic Mutations and DTL Expression in HCC

**Figure 5:**
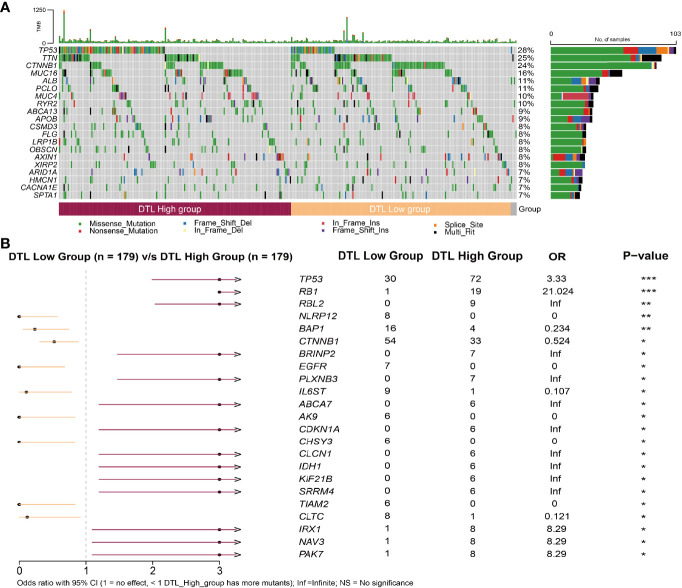
Relationship between somatic mutations and DTL expression in HCC. **(A)** Somatic mutations in DTL-high and DTL-low expression groups. **(B)** Comparison of mutations between the high expression group and low expression group of DTL. *P < 0.05, **P < 0.01, ***P < 0.001.

We collected the mutation profiles from the HCC cohort in the TCGA database to explore whether the distribution of mutations in the HCC cohort is affected by the expression of the DTL gene, in consideration of the crucial role of the *DTL* gene in cell proliferation. We profiled the mutation map of patients on two groups (DTL^high^ vs. DTL^low^ group) (**Figure 5A**) and found that, different from the top 5 gene of mutations in the low expression group [*CTNNB1* (30%), *TTN* (25%), *TP53* (17%), *MUC16* (14%), *PCLO* (12%)], the genes with a higher proportion of mutations in the DTL^high^ group are *TP53* (40%), *TTN* (25%), *CTNNB1* (18%), *MUC16* (18%), and *MUC4* (12%) (**Supplementary Figures 5A, B**). We noted that patients in the DTL^high^ group showed a higher rate (40%) of *TP53* mutation, and the difference was statistically significant (**Figure 5B**). The distribution of patients with *CTNNB1* mutation was also different in terms of DTL expression, which was different from the case of *TP53* mutation. The patients with low-DTL expression possessed a higher rate (30%) of *CTNNB1* mutation (**Figure 5B** and **Supplementary Figure 5B**). In addition, we also noted that in the comparison of mutations in the high and low expression groups, most genes had more mutations in the high DTL expression group (**Figure 5B**). This might be associated with the high amplifications of HCC cells with DTL overexpression phenotype and the resulting DNA replication errors.

### The Relationship of DTL Expression With Marker Gene Sets of Immune Cells

**Figure 6:**
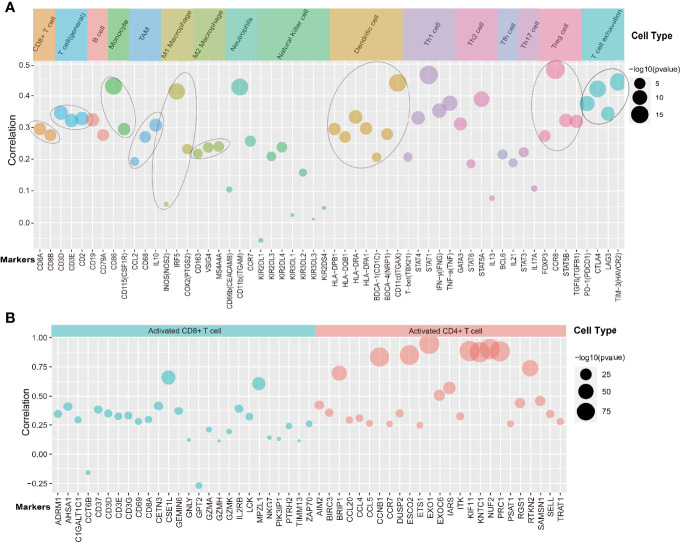
The relationship of DTL expression with marker gene sets of immune cells. **(A, B)** The bubble plot shows the correlations of the DTL gene with marker sets of 16 diverse immune cells or their status **(A)**, as well as markers of activated CD8+/CD4+ T cells **(B)** summarized from the Timer 2.0 database.

Our above findings showed that high levels of the DTL gene in cells would lead to rapid tumor progression; however, how the behavior of pro-proliferation characteristics of HCC cells affects the microenvironment is unknown. Therefore, we sought to elucidate the relevance of DTL expression with the status of diverse infiltrating immune cells. Interestingly, the close association of the DTL gene and markers of Treg cells and T cell exhaustion, such as FOXP3, CCR8, STAT5B, PD-1, CTLA4, and LAG3, was found after adjusting the correlation values for tumor purity (**Figure 6A**). The expression of FOXP3 in Treg cells hindered the body’s immune system from screening abnormal cells, hence creating an environment for malignant cells to proliferate, so the phenomenon of immune escape occurred (19). PD-1/PD-L1 binding could transmit inhibitory signals in T cells, leading to the failure of tumor-associated T cells to activate, causing the reduction or even failure of antitumor immunity. Other T cell exhaustion markers, such as CTLA4 and LAG3, were also closely related with *DTL* levels (**Figure 6A**). This evidence hinted the close association of *DTL* levels with the exhausted status of T cells and that *DTL* might play a crucial role in immune escape in the microenvironment of liver cancer. Meanwhile, markers of CD8^+^ T cells and T cells (general) showed positive correlations with *DTL* expression (**Figure 6A**). To examine the association between the status of T cells and DTL expression, the associations of *DTL* levels with markers of T-cell activation, which included marker genes of activated CD8^+^ T cells and CD4^+^ T cells (**Figure 6B**), were also analyzed. After balancing the impact of tumor purity, the mRNA levels of the *DTL* gene strongly correlated with most markers of activated CD8^+^ and CD4^+^ T cells in HCC. In addition, the markers of TAMs, M1 macrophages, M2 macrophages, and monocytes had strong associations with *DTL* levels (**Figure 6A**). We also validated the correlations between *DTL* levels and the markers of monocytes and tumor-associated macrophages (TAMs) *via* the GEPIA database. The relevance of *DTL* levels with gene sets of monocytes and TAMs was consistent with those conducted *via* TIMER (**Supplementary Table S3**). This evidence suggested that the *DTL* gene affected the status of macrophage polarization in liver cancer.

### Characteristics of Immune Cell Infiltration in Tumors With DTL High Expression

**Figure 7:**
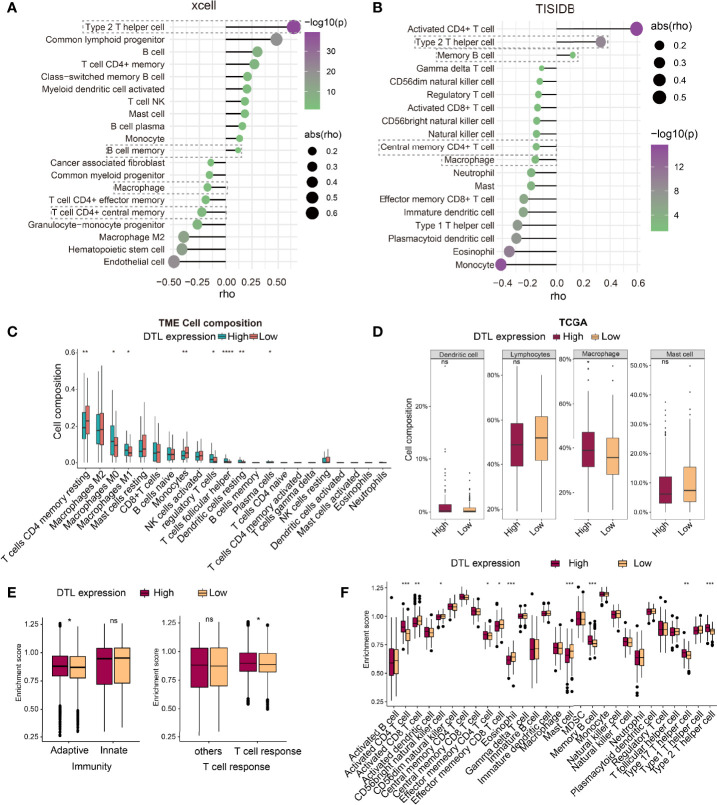
Characteristics of immune cell infiltration in tumors with DTL high expression. **(A, B)** The relationship between DTL expression and immune cell infiltrates was analyzed by xcell and TISIDB platforms. **(C)** Proportions of 22 immune cells in subgroups (DTL^high^ and DTL^low^) of HCC. **(D)** Box plot shows differences in composition of four types of immune cells in the high- and low-*DTL* groups. **(E)** Differences between the activities of adaptive immunity, innate immunity, and T cell response in high-/low- DTL expression groups. **(F)** Differences in the activities of diverse immune cells between high and low expression groups of the DTL gene. *P < 0.05, **P < 0.01, ***P < 0.001, ****P < 0.0001. ns, No significance.

While we had explored the relevance of DTL expression with markers of immune cells or their status, the characteristics of immune cell infiltration in HCC tumors with high DTL expression remained unknown. Therefore, we next investigated the relevance of DTL levels with the infiltrating abundance of various immune cells, which was based on different databases (xcell and TISIDB), in liver cancer. The results of both algorithms hinted that *DTL* levels were positively related with the infiltration of type 2 T helper cells and memory B cells in HCC (**Figures 7A, B**). However, DTL levels showed a negative correlation with the infiltrating levels of macrophages and central memory CD4+ T cells (**Figures 7A, B**). Next, to further understand the relationship between the levels of DTL gene and the composition of diverse immune cells in the tumor immune microenvironment (TME) of HCC, we applied the CIBERSORT algorithm to dissect the TME of HCC patients with different expressions (high and low) of *DTL*. The relative proportions of the 22 different immune cells have been characterized, as shown in **Figure 7C**. The plot revealed that in the TME of HCC patients, the top four proportions of cells are CD4+ T cells (memory resting), macrophages M2, macrophages M0, and mast cells resting (**Figure 7C**). Moreover, patients with a high-DTL expression have relatively higher proportions of macrophages M0 and macrophages M1 than those with a low DTL expression, suggesting that the rapid proliferation of highly DTL-expressed cells in tumors leads to inflammatory response (**Figure 7C**). At the same time, the TME of tumors high *DTL* expression demonstrated to be suppressive, as indicated by the high infiltration of Treg cells and relatively low infiltration of CD8+ T cells (**Figure 7C**). In addition, these 22 types of immune cells are also divided into four categories, including dendritic cells, lymphocytes, macrophages, and mast cells. The differences in infiltration of four categories in terms of DTL expression (high and low) were also calculated. The results reveal that the infiltration of macrophages was relatively high in the DTL^high^ group while the proportions of other three categories did not show differences between two groups (**Figure 7D**). The activities of immune cells were further analyzed by using mRNA expression profiling of the TCGA-HCC cohort *via* single-sample gene set enrichment analysis (ssGSEA, **Supplementary Figure 6A**). The analyzed immune cell activities were divided into two subtypes (antitumor immunity and pro-tumor, immune-suppressive functions) based on the function of immune cells in TME (20). However, the high-DTL expression group in the HCC cohort did not show heterogeneity with the low-DTL expression group in promoting tumor activity and antitumor activity (**Supplementary Figure 6B**). Interestingly, tumors with a high expression of the *DTL* gene would inflame the relative strong adaptive immunity within them, but there is no difference when they came to the innate immunity (**Figure 7E**, left). In terms of adaptive immunity, tumors with a DTL overexpression phenotype would cause a relatively strong T cell response (**Figure 7E**, right), although the responses mainly came from the activity of CD4 + T cells. The activity of CD8 + T cells, whether the activated or effector memory ones, is lower than that in tumors with low DTL expression (**Figure 7F**).

## Discussion

Cell division is a key step in tumor growth, leading to the production of two similar clones, which is of great significance for tumor growth and tumorigenesis. Usually combined with the cullin ring E3 ubiquitin ligase (CRL) to form a complex (CRL^CDT2^), DTL (CDT2) mainly plays a pivotal role in genomic stability within tumor cells, which is important for tumor cells to duplicate. To pinpoint the potential role of DTL in HCC and its subtle impact in TME, we conducted a systematical analysis on public data. Exploration of the transcriptome in specimens from 5 GEO datasets and the HCC cohort from both TCGA and ICGC databases proved that *DTL* expression is significantly overexpressed in tumor tissues than non-tumor specimens (**Figure 1**). KEGG enrichment, GO analysis, and GSEA results demonstrated that the tumor cells of patients with the *DTL*-overexpression phenotype were highly active in the process of cell cycle, DNA replication, and cell division. This also explains why the overexpression DTL phenotype in the HCC cohort is significantly correlated with these clinical features (T stages and pathological stage), and patients whose tumors overexpressed the DTL gene, accompanying a higher proportion of somatic mutations (e.g., *TP53*), had much worse clinical outcomes than those whose tumors did not across HCC. Furthermore, multivariate Cox regression analysis validated that the upregulated DTL levels in patients are an independent predictor for unfavorable prognosis, suggesting that the DTL gene had a prognostic value as a biomarker for HCC.

The function of the complex composed of CDT2(DTL) and CRL4 is special, which is related to DNA synthesis and genomic stability. Their substrates include CDT1, CDK1A, and KMT5A, key regulators of DNA replication (21). In tumor cells, when DNA is damaged, the degradation of these proteins can ensure the termination of DNA replication, so as to protect the stability of their genome and ensure the survival of cells. Knockout of CDT2 could lead to serious genomic instability, while overexpression of CDT2 would cause cells to reenter the cell cycle (22). Therefore, CDT2 promotes accurate DNA replication by the temporal regulation of replication licensing, which is required for cell proliferation and development (23–25). Hence, the DTL (CDT2) gene might be a potential therapeutic target. Inhibiting CRL4^CDT2^ by CDT2 knockdown could be an effective way in eliminating the cells of cutaneous squamous cell carcinoma (cSCC) than targeting CRL4 alone due to its role in cell-cycle progression and genomic stability, and loss or low levels of DTL would lead to serious DNA damage (26). Not just HCC alone, DTL (CDT2) is also highly expressed in cutaneous melanoma and reflects unfavorable OS and DFS. CDT2 is an indispensable molecule for melanoma cells to proliferate and inhibit the CRL4^CDT2^ complex by pevonedistat [a specific inhibitor of the NEDD8-activating enzyme (NAE)], which attenuates the function of cullin E3 ligases and inhibits melanoma by inducing DNA re-replication and senescence through the stabilization of p21 and SET8, the CRL4^CDT2^ substrates (27). Vanessa et al. found that DTL (CDT2) expression is augmented in head and neck squamous cell carcinoma (HNSCC) and knockdown of DTL by siRNA attenuated the growth of human papillomavirus negative (HPV-ve) HNSCC cells. Applying the NAE inhibitor, pevonedistat (MLN4924), in the treatment of HNSCC induces remarkable re-replication and attenuates HNSCC cell proliferation in culture and mouse models (9). We retrospectively analyzed the cases of MLN4924 treatment included in the GEO database (GSE184850 and GSE30531) and found that after the treatment, the expression of DTL would reactively increase and lead to the inhibition of cell cycle- and DNA replication-related genes, while the expression of apoptosis-related genes increased in two tumor cell lines (**Supplementary Figure 7**). Together, these previous studies are partially consistent with the pan-cancer overexpression characteristics of DTL in our study and suggest that pevonedistat (MLN4924) may be the targeted therapy in treatment of HCC cases.

Our findings also revealed that *DTL* expression was related with the infiltrating abundance of various immunocytes in HCC, which had not been comprehensively explored so far. Results indicated that *DTL* levels were strongly related with the infiltrating levels of type 2 T helper cells and memory B cells in HCC and negatively associated with central memory CD4^+^ T cells and macrophages. Further analysis demonstrated that transcriptional levels of the DTL gene were closely related with markers of CD8^+^ T cells, T cells (general), monocytes, DC cells, Treg cells, and T cell exhaustion in HCC. Interestingly, our data showed that the *DTL* expression showed positive associations with most markers of activated T cells, specifically CD8^+^/CD4^+^ T cells, in liver cancer, suggesting that HCC tumors with the overexpression DTL phenotype might potentially be a hot tumor. CD8^+^ T cells, as the leading antitumor T cells, would eliminate malignant cells by releasing perforin and granzyme B through the Fas/FasL pathway as soon as they touch cancerous cells or release IFN-γ and TNFα to destroy them (28), although these cells in the TME usually demonstrated to be functionally exhausted, which is closely linked to the expression of immune checkpoints (e.g., PD1 and CTLA4), as is demonstrated in this study. Most malignancies, HCC included, augment the levels of inhibitory ligands to avoid immune response by attenuating the function of T cells, thus leading to tumor growth. This is considered to be one of the potential mechanisms facilitating tumor progression, implying that patients with high DTL expression may derive greater OS benefit from immune checkpoint inhibitor- (ICI-) based therapy in the clinical setting. Moreover, the levels of Treg cell markers, say, FOXP3, TGF-β, and CCR8, showed positive correlations with the expression of *DTL* levels. Previous studies show that as the tumor progresses, the number of Treg cells, the crucial immunosuppressive cells, increases (29), while, in our study, Treg cells infiltrate more in the DTL^high^ group, hinting that patients with *DTL* overexpression might show some degrees of immunosuppression. TAMs could help malignant cells in different ways, including promoting angiogenesis, immune escape, and metastasis (30–33), while, in our studies, there exist strong relationships of *DTL* expression levels with marker gene sets of TAMs in HCC, suggesting that the tumor progression in hepatic DTL-overexpressed patients might have something to do with the TAMs.

Although our evidence implies that *DTL* is a cancer-related gene, there exist some limitations in our study. Our explorations into the role of *DTL* in HCC were based on preexisting data from the GEO, TCGA, and TIMER databases, and some of our findings were verified by our validation cohort; however, we did not conduct *in vivo* and *in vitro* experiments to confirm the function of *DTL* in the biological processes of the cell cycle and DNA replication or its relationship with the infiltrating immune cells in TME, which pinpoint the direction for our future work.

In conclusion, this study reveals that the *DTL* gene is an underlying prognostic biomarker for patients with HCC and is related with the infiltrating levels of immune cells in the tumor microenviroment. HCC patients with a DTL overexpression phenotype may incur a greater risk of tumor advance, and in view of the diversity in infiltrating levels and status of immune cells between the groups of high- and low-DTL expression, patients with DTL overexpression may gain benefits from more precise immunotherapy strategies in the clinics.

## Data Availability Statement

The datasets presented in this study can be found in online repositories. The names of the repository/repositories and accession number(s) can be found in the article/**Supplementary Material**.

## Ethics Statement

The studies involving human participants were reviewed and approved by the Ethical Committees of Shanghai General Hospital. The patients/participants provided their written informed consent to participate in this study.

## Author Contributions

YS and ZL conceived and designed the study. ZL analyzed the acquired data. RW supervised the integral analysis. CQ, CC, JG, and JZ contributed to the data collection, curation, and interpretation. All authors contributed to the article and approved the submitted version.

## Conflict of Interest

The authors declare that the research was conducted in the absence of any commercial or financial relationships that could be construed as a potential conflict of interest.

## Publisher’s Note

All claims expressed in this article are solely those of the authors and do not necessarily represent those of their affiliated organizations, or those of the publisher, the editors and the reviewers. Any product that may be evaluated in this article, or claim that may be made by its manufacturer, is not guaranteed or endorsed by the publisher.

## Supplementary Material

Supplementary Figure 1 The Up-regulated *DTL* expression in various tumor types. **(A)** Transcriptional expression of *DTL* in different tumor types from the TCGA database determined by TIMER using the Wilcoxon test. **P* < 0.05, ***P* < 0.01, ****P* < 0.001, *****P* < 0.0001.

Supplementary Figure 2 High expression of DTL predicts worse prognosis in HCC. **(A, B)** Disease specific survival (DSS) analysis of the effect of DTL gene on HCC cohort subgrouped by gender, ethnics, alcohol abuse, and infection of hepatitis virus.

Supplementary Figure 3
*DTL* is vital in cell cycle and DNA replication of HCC cells. **(A, B)** KEGG pathway enrichment and GO analysis of 581 down-regulated DEGs. **(C–E)** GSEA analysis prompts *DTL* is positively related to choromosome segregation, DNA replication initiation, and P53 signaling pathway.

Supplementary Figure 4 Associations between *DTL* and key genes related to cell cycle and DNA replication. **(A–F)** The impact of DTL knockdown on the expression of apotosis-, cell cycle- and DNA replication-related genes on three Ewing’s sarcoma (ES) cell lines.

Supplementary Figure 5 Relationship between somatic mutations and DTL expression in HCC. **(A, B)** The mutation patterns of patients with high- or low- DTL expression were analyzed respectively.

Supplementary Figure 6 Analysis of the activity of infiltrating immune cells in DTL-high/low expression groups in HCC. **(A)** ssGSEA analysis identifying the activities of immune cells for 371 HCC-tumor samples with available RNA-seq data from TCGA database.** (B)** Analysis of the activity of pro-tumor suppression and anti-tumor immunity in high- and low-*DTL* group.

Supplementary Figure 7 The exploration of therapeutic potential of MLN4924 on tumors. **(A–E)** The impact of MLN4924 on the expression of apotosis-, cell cycle- and DNA replication-related genes on two cell lines.
