# Supplementary figures and images for: Role of DTL in Hepatocellular Carcinoma and Its Impact on the Tumor Microenvironment
Source: Front Immunol. 2022 Mar 22;13:834606. doi: 10.3389/fimmu.2022.834606 (PMC8980229; doi:10.3389/fimmu.2022.834606)

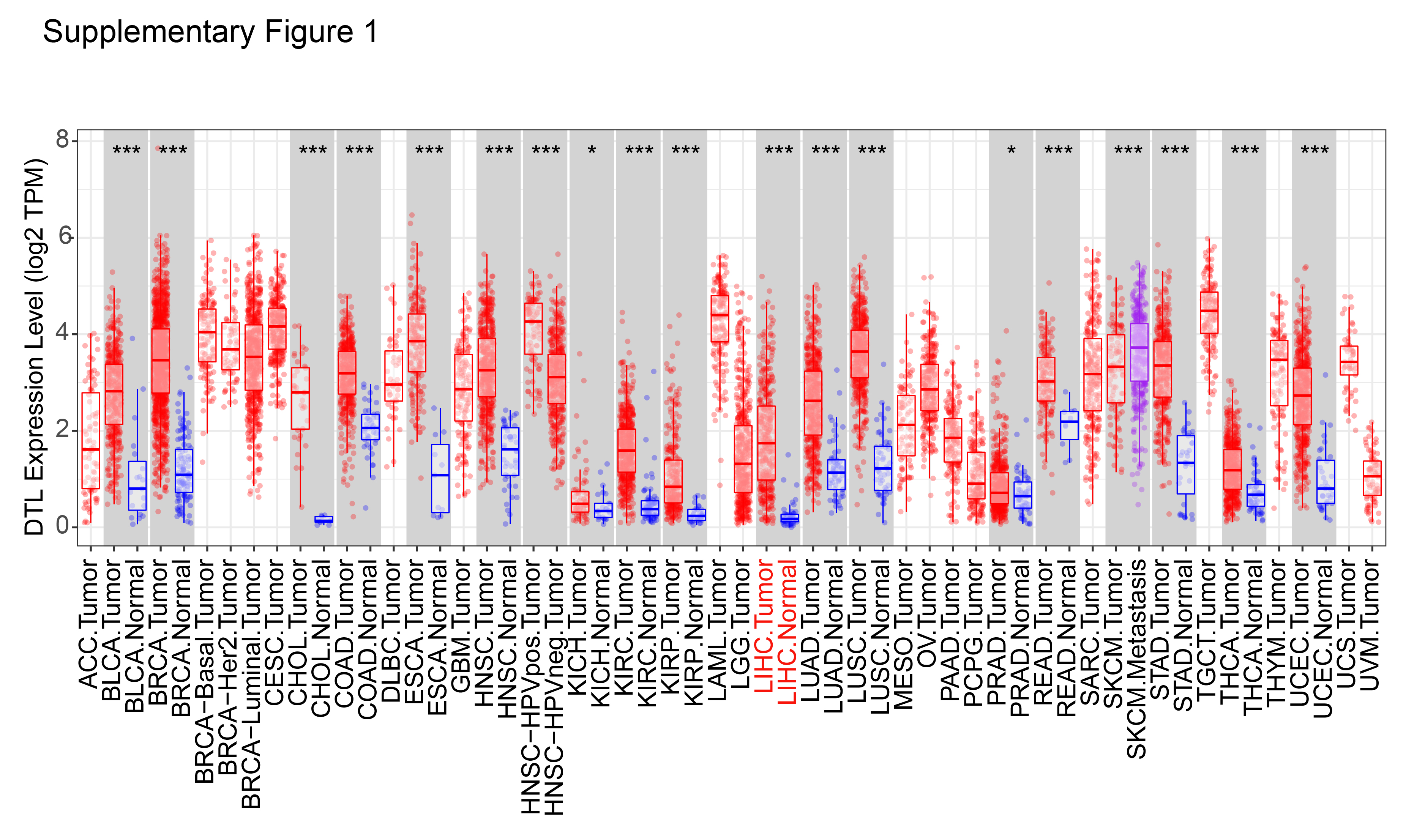

Supplement: Supplementary Figure 1 — The Up-regulated DTL expression in various tumor types. (A) Transcriptional expression of DTL in different tumor types from the TCGA database determined by TIMER using the Wilcoxon test. *P < 0.05, **P < 0.01, ***P < 0.001, ****P < 0.0001. [file Image_1.jpg]

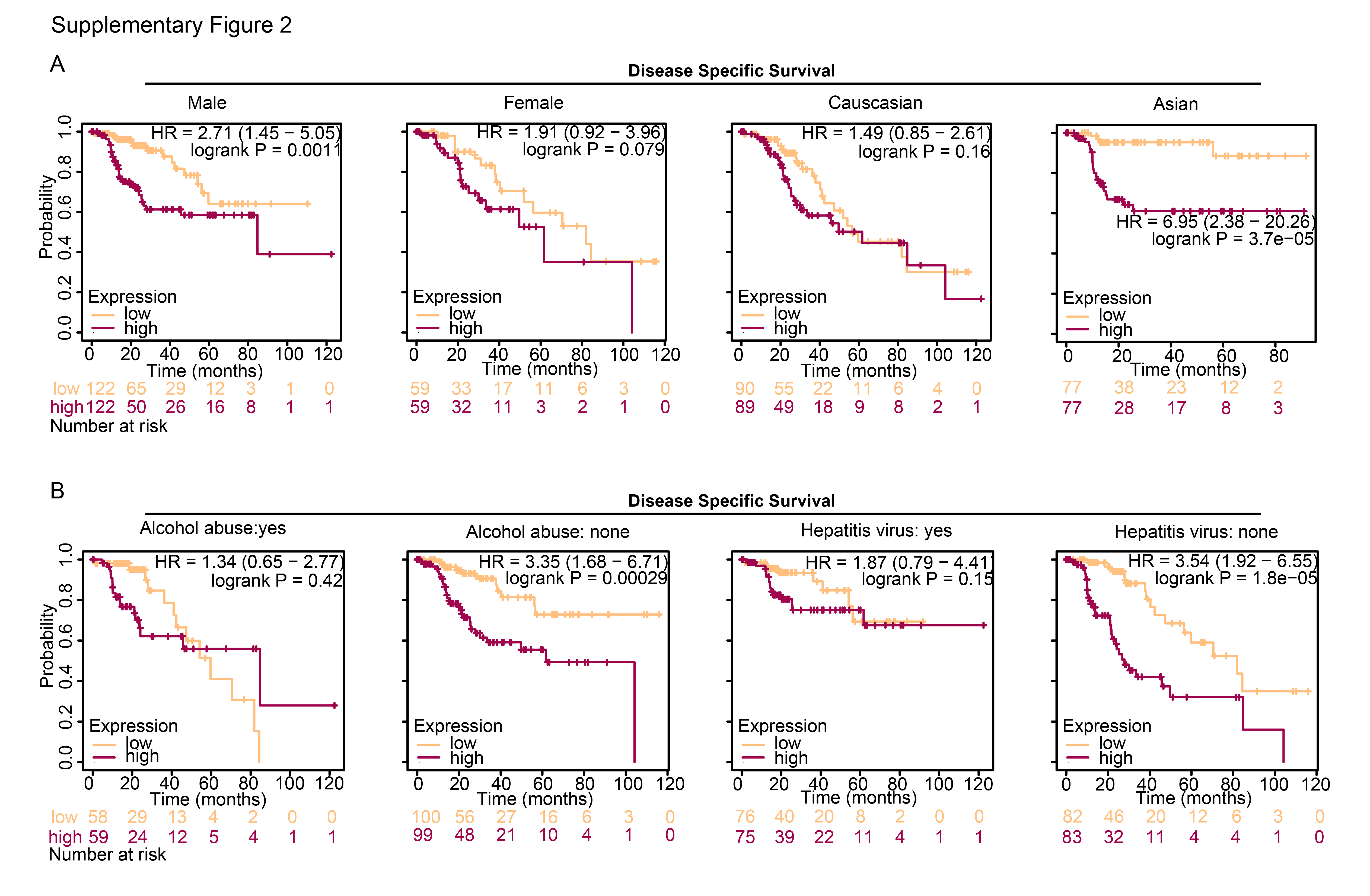

Supplement: Supplementary Figure 2 — High expression of DTL predicts worse prognosis in HCC. (A, B) Disease specific survival (DSS) analysis of the effect of DTL gene on HCC cohort subgrouped by gender, ethnics, alcohol abuse, and infection of hepatitis virus. [file Image_2.jpeg]

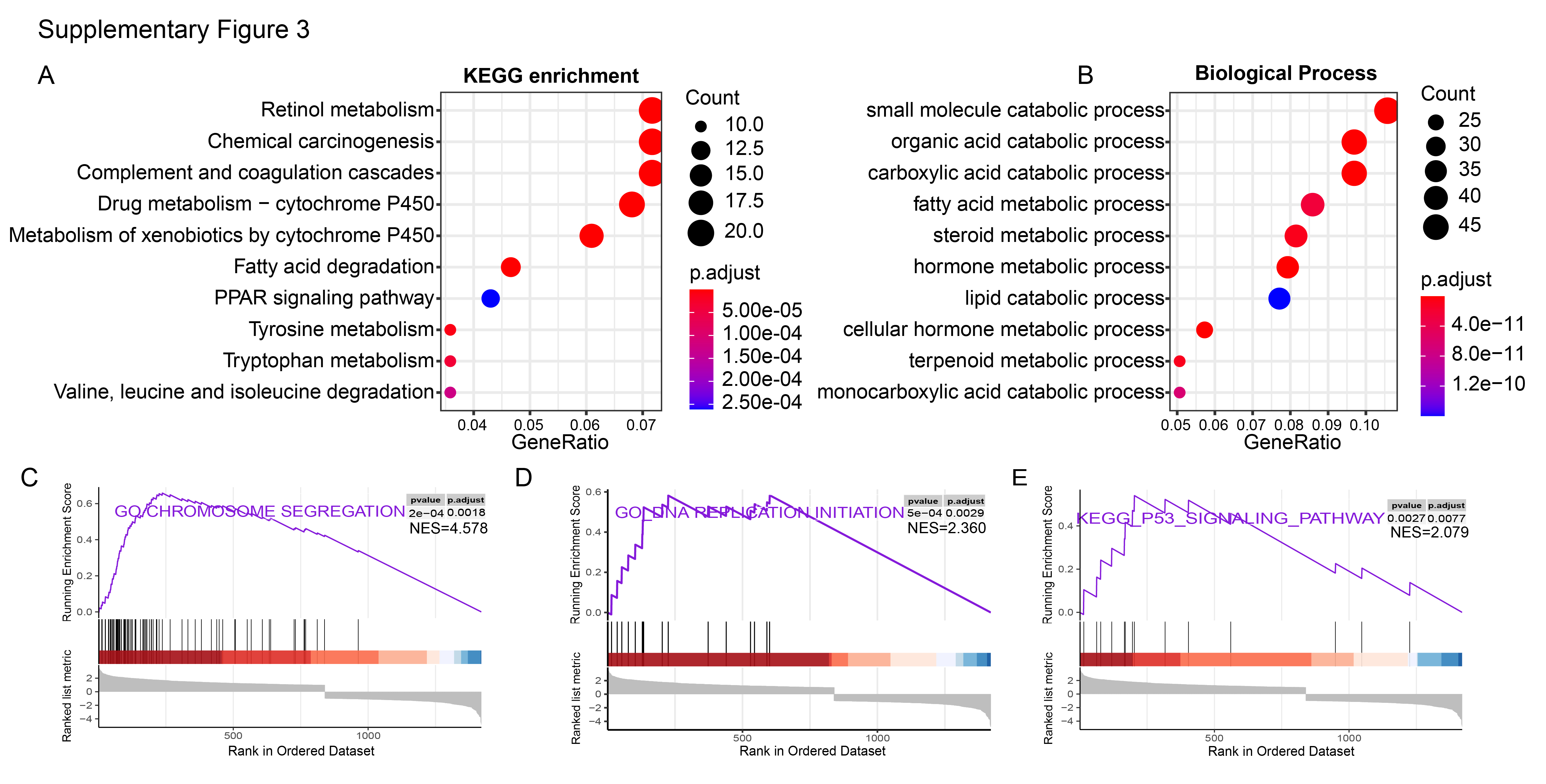

Supplement: Supplementary Figure 3 — DTL is vital in cell cycle and DNA replication of HCC cells. (A, B) KEGG pathway enrichment and GO analysis of 581 down-regulated DEGs. (C–E) GSEA analysis prompts DTL is positively related to choromosome segregation, DNA replication initiation, and P53 signaling pathway. [file Image_3.jpeg]

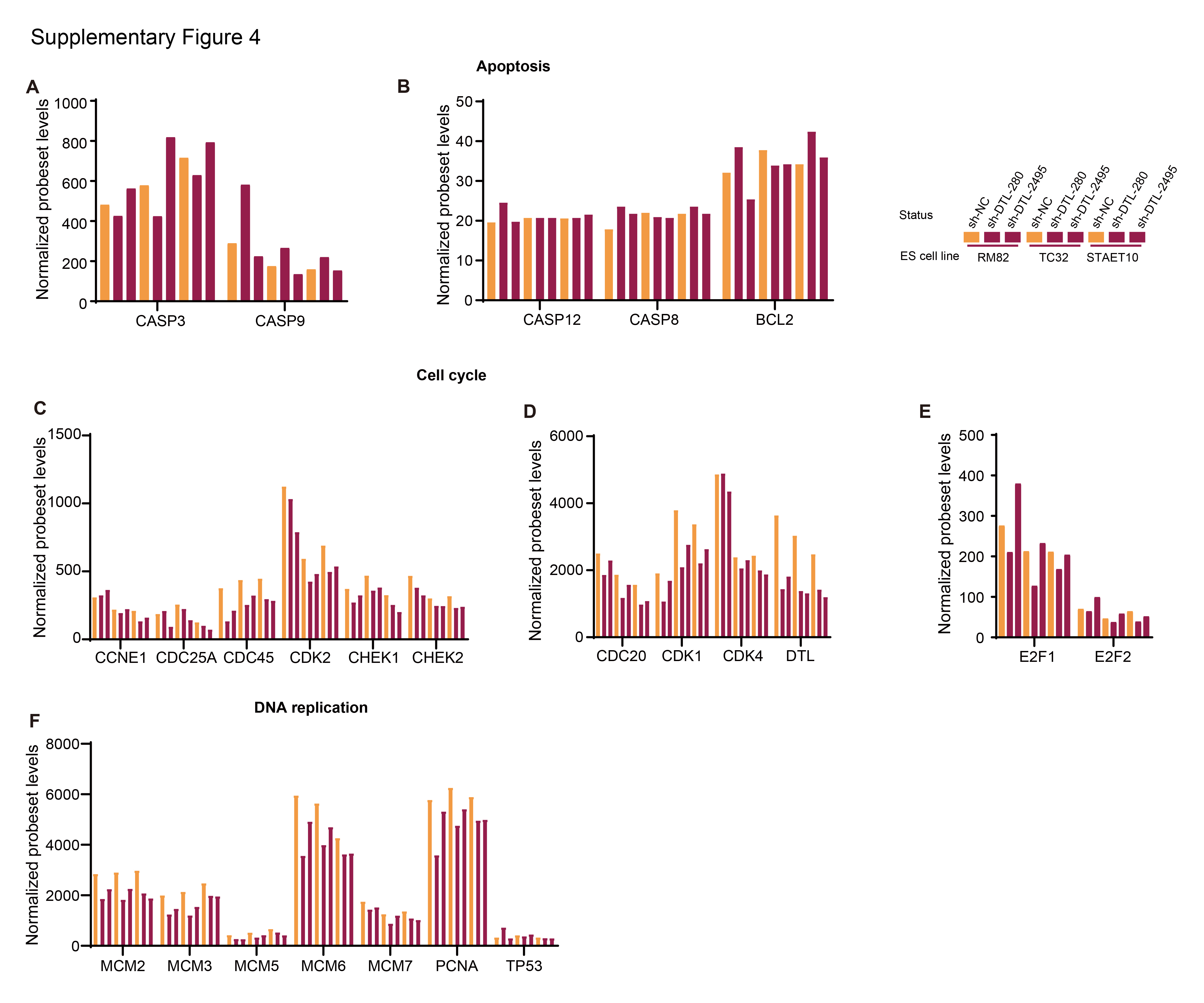

Supplement: Supplementary Figure 4 — Associations between DTL and key genes related to cell cycle and DNA replication. (A–F) The impact of DTL knockdown on the expression of apotosis-, cell cycle- and DNA replication-related genes on three Ewing’s sarcoma (ES) cell lines. [file Image_4.jpeg]

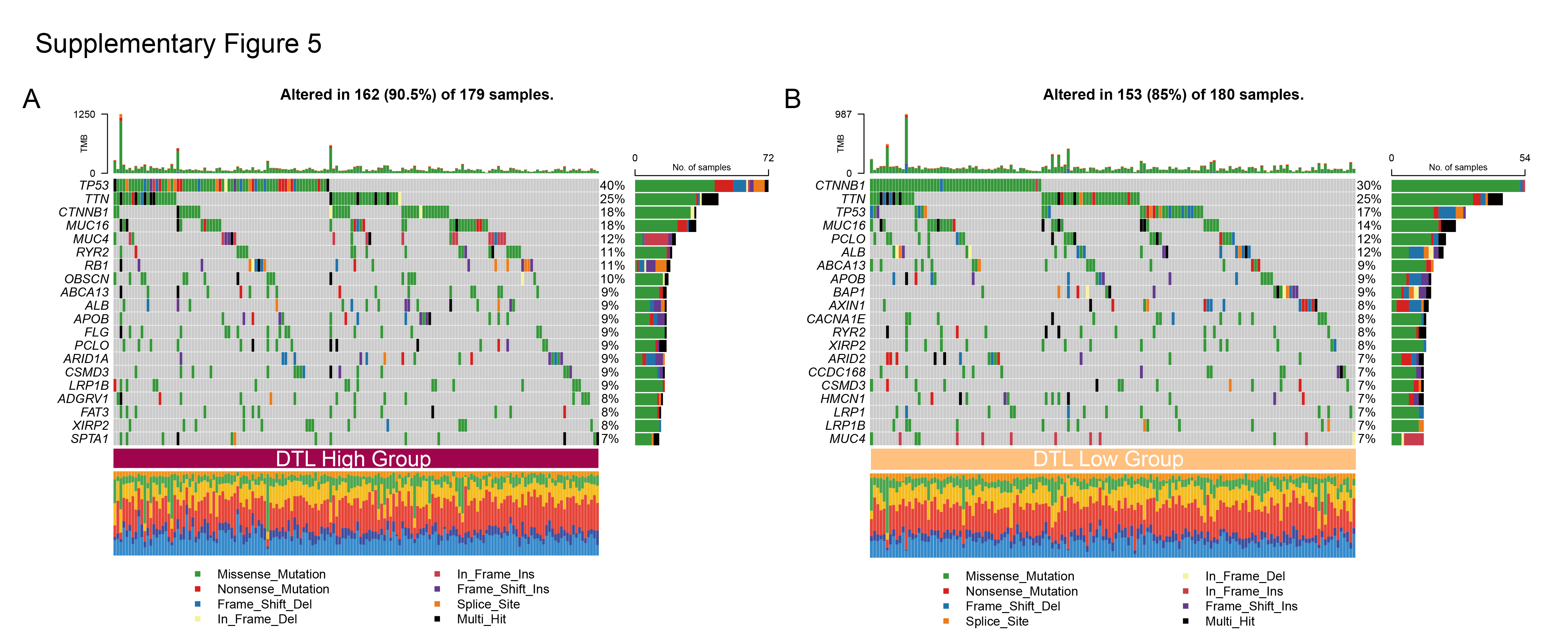

Supplement: Supplementary Figure 5 — Relationship between somatic mutations and DTL expression in HCC. (A, B) The mutation patterns of patients with high- or low- DTL expression were analyzed respectively. [file Image_5.jpeg]

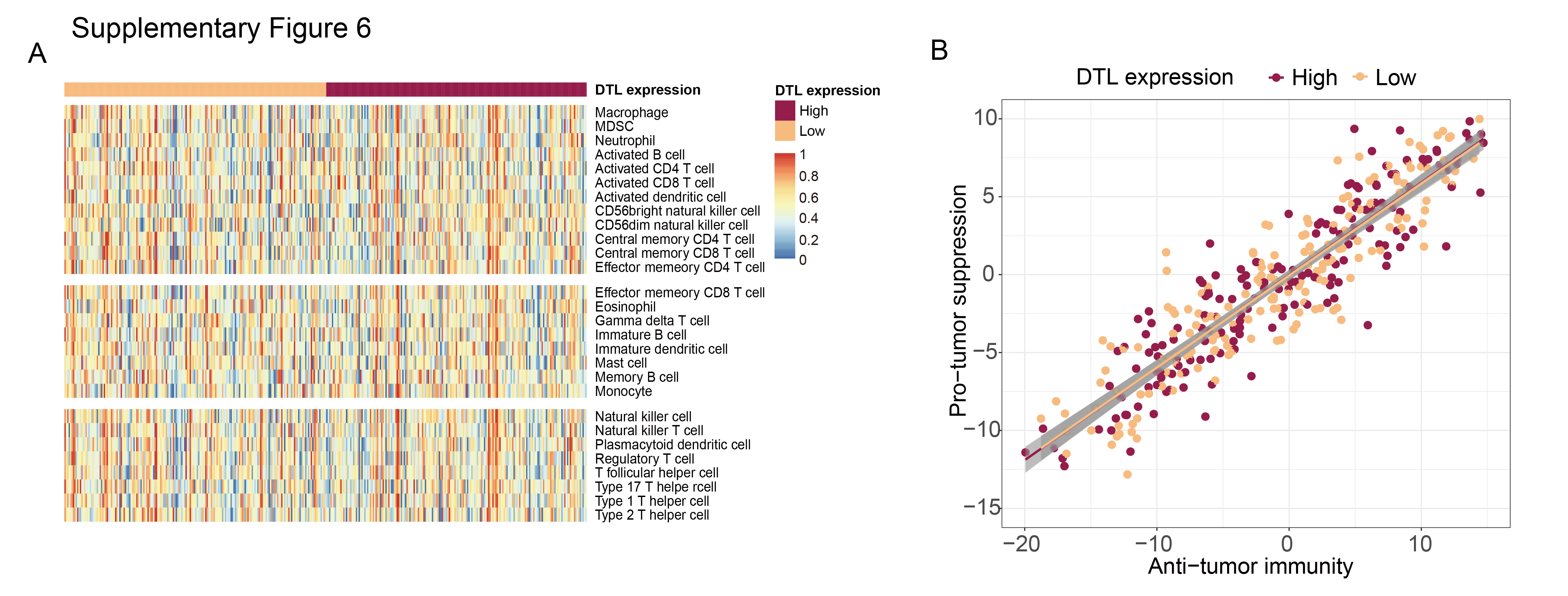

Supplement: Supplementary Figure 6 — Analysis of the activity of infiltrating immune cells in DTL-high/low expression groups in HCC. (A) ssGSEA analysis identifying the activities of immune cells for 371 HCC-tumor samples with available RNA-seq data from TCGA database. (B) Analysis of the activity of pro-tumor suppression and anti-tumor immunity in high- and low-DTL group. [file Image_6.jpeg]

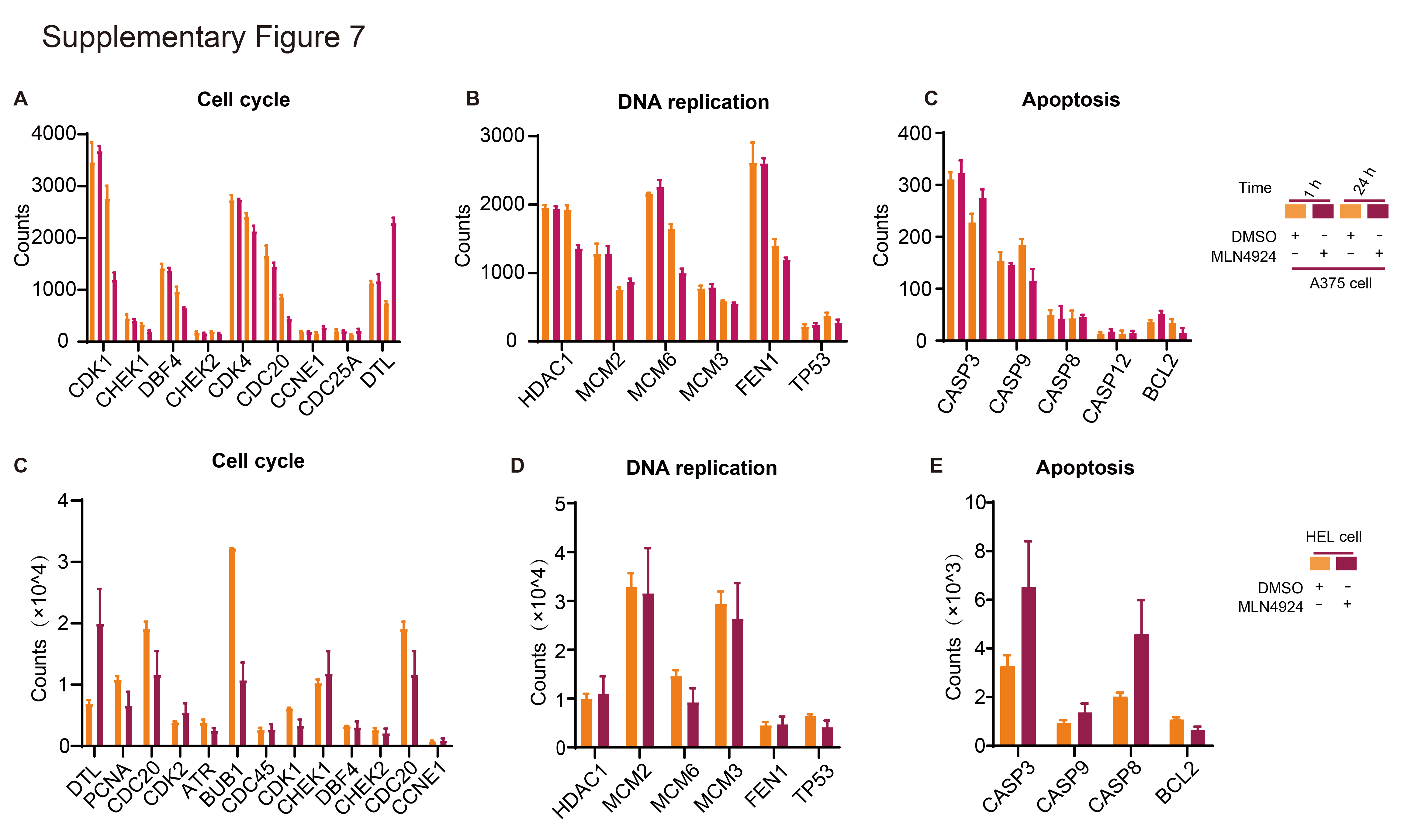

Supplement: Supplementary Figure 7 — The exploration of therapeutic potential of MLN4924 on tumors. (A–E) The impact of MLN4924 on the expression of apotosis-, cell cycle- and DNA replication-related genes on two cell lines. [file Image_7.jpeg]
