# Supplementary material for: Role of DTL in Hepatocellular Carcinoma and Its Impact on the Tumor Microenvironment
Source: Front Immunol. 2022 Mar 22;13:834606. doi: 10.3389/fimmu.2022.834606 (PMC8980229; doi:10.3389/fimmu.2022.834606)
Supplement: Supplementary file 8 [file Table_1.docx]

| GEO series | Contributors | Nontumor | Tumor | Platform |
| --- | --- | --- | --- | --- |
| GSE77314 | Liu G, Hou G, Li L, Li Y et al.2016 | 50 | 50 | Illumina Genome Analyzer (Homo sapiens) |
| GSE45436 | Wang HW, Hsieh TH, Chau GY et al. 2013 | 39 | 95 | Affymetrix Human Genome U133 Plus 2.0 Array |
| GSE36376 | Lim HY, Sohn I, Deng S, Lee J et al.2013 | 193 | 249 | Illumina HumanHT-12 V4.0 expression beadchip |
| GSE25097 | Zhang C.et al.2011 | 243 | 268 | Rosetta/Merck Human RSTA Affymetrix 1.0 microarray, Custom CD |
| GSE14520 | Roessler S, Jia HL, Forgues M et al.2010 | 220 | 225 | Affymetrix HT Human Genome U133A Array |

**Supplementary Table 1. Details of the five cohorts used in this study.**
