# Supplementary material for: Role of DTL in Hepatocellular Carcinoma and Its Impact on the Tumor Microenvironment
Source: Front Immunol. 2022 Mar 22;13:834606. doi: 10.3389/fimmu.2022.834606 (PMC8980229; doi:10.3389/fimmu.2022.834606)
Supplement: Supplementary file 9 [file Table_2.docx]

**Supplementary Table 2. Clinicopathological characteristics in relation to DTL expression status in the validation cohort.**

|  | Case  (N=114) | IHC score | |  |  |
| --- | --- | --- | --- | --- | --- |
| **characteristics** |  | <6 | >=6 | χ2 | *P* value |
| **Age** |  |  |  |  |  |
| >=60y |  | 0(0) | 14(100) |  | **<0.001^a^** |
| <60y |  | 52(52) | 48(48) |  |  |
| **Gender** |  |  |  |  |  |
| male |  | 45（45） | 55（55） | 0.124 | 0.725 |
| female |  | 7（50） | 7（50） |  |  |
| **History of hepatitis** |  |  |  |  |  |
| Yes |  | 40(48.8) | 42(51.2) | 1.181 | 0.277 |
| no |  | 12(37.5) | 20(62.5) |  |  |
| **Status** |  |  |  |  |  |
| Live |  | 52(69.3) | 23(30.7) |  | **<0.001^a^** |
| dead |  | 0(0) | 39(100) |  |  |
| **Tumor size** |  |  |  |  |  |
| < 5cm |  | 41(80.4) | 10(19.6) | 44.995 | **<0.001** |
| >= 5cm |  | 11(17.5) | 52(82.5) |  |  |
| **Tumor Thrombus** |  |  |  |  |  |
| Yes |  | 22(43.1) | 29(56.9) | 0.096 | 0.757 |
| none |  | 29(46) | 34(54) |  |  |
| **T** |  |  |  |  |  |
| T1-T2 |  | 42(46.7) | 48(53.3) | 0.644 | 0.422 |
| T3-T4 |  | 9(37.5) | 15(62.5) |  |  |
| **N** |  |  |  |  |  |
| N0 |  | 51(46.4) | 59(53.6) |  | 0.127^a^ |
| N1 |  | 0(0) | 4(100) |  |  |
| **M** |  |  |  |  |  |
| M0 |  | 55(44.6) | 62(55.4) | 0.000 | 1 |
| M1 |  | 1(50) | 1(50) |  |  |

Statistical significance was determined by Chi-square test (if necessary, results were adjusted by Yate’s correction) or Fisher's exact test (^a^).
