# Supplementary material for: Role of DTL in Hepatocellular Carcinoma and Its Impact on the Tumor Microenvironment
Source: Front Immunol. 2022 Mar 22;13:834606. doi: 10.3389/fimmu.2022.834606 (PMC8980229; doi:10.3389/fimmu.2022.834606)
Supplement: Supplementary file 10 [file Table_3.docx]

**Supplementary Table 3. Correlation analysis between DTL and related genes and markers of monocyte and macrophages in GEPIA.**

| Description | Gene markers | LIHC | | | |
| --- | --- | --- | --- | --- | --- |
|  |  | Tumor | | Normal | |
|  |  | R | *P* | R | *P* |
| Monocyte | CD86 | 0.250 | 1.20E-06* | 0.400 | 4.50E-03* |
|  | CD115(CSF1R) | 0.150 | 3.60E-02* | 0.400 | 4.30E-03* |
| TAM | CCL2 | 0.049 | 3.50E-01 | 0.120 | 4.00E-01 |
|  | CD68 | 0.170 | 8.20E-04* | 0.320 | 2.40E-02* |
|  | IL10 | 0.120 | 1.70E-02* | 0.200 | 1.60E-01 |
| M1 Macrophage | INOS(NOS2) | 0.100 | 4.90E-02* | 0.180 | 2.20E-01 |
|  | IRF5 | 0.380 | 1.90E-14* | 0.200 | 1.70E-01 |
|  | COX2(PTGS2) | 0.110 | 3.70E-02* | 0.120 | 4.10E-01 |
| M2 Macrophage | CD163 | 0.023 | 6.50E-01 | 0.280 | 5.20E-02 |
|  | VSIG4 | 0.110 | 4.30E-02* | 0.270 | 5.40E-02 |
|  | MS4A4A | 0.087 | 9.40E-02 | 0.360 | 9.90E-03* |

R. Value of Spearman’s correlation; *P*. Statistical significance. * *P* < 0.05.
